# Functional intratumoral lymphatics in patient-derived xenograft models of squamous cell carcinoma of the uterine cervix: implications for lymph node metastasis

**DOI:** 10.18632/oncotarget.10931

**Published:** 2016-07-29

**Authors:** Einar K. Rofstad, Ruixia Huang, Kanthi Galappathi, Lise Mari K. Andersen, Catherine S. Wegner, Anette Hauge, Jon-Vidar Gaustad, Trude G. Simonsen

**Affiliations:** ^1^ Group of Radiation Biology and Tumor Physiology, Department of Radiation Biology, Institute for Cancer Research, Oslo University Hospital, Oslo, Norway

**Keywords:** cervix carcinoma, patient-derived xenografts, lymphangiogenesis, metastasis, interstitial fluid pressure

## Abstract

Studies of cell line-derived human tumor xenografts have suggested that the lymphatics seen in immunohistochemical preparations from non-peripheral regions of tumors are nonfunctional. In this investigation, lymphangiogenesis, hemangiogenesis, and lymph node metastasis were studied in patient-derived xenograft (PDX) models of carcinoma of the uterine cervix. Lymph vessel density (LVD) and blood vessel density (BVD) were measured in immunohistochemical preparations. The expression of angiogenesis-related genes was investigated by quantitative PCR. Lymphatic functionality was assessed with the ferritin assay, and tumor interstitial fluid pressure (IFP) was measured with a Millar catheter. The PDX models mirrored the angiogenesis and aggressiveness of the donor patients' tumors, and two highly aggressive models developed functional lymphatics within the tumor mass. Tumors with functional intratumoral lymphatics showed low IFP, high LVD, high BVD, high expression of a large number of angiogenesis-related genes, and high incidence of lymph node metastases. LVD correlated with BVD, and lymph node metastasis was associated with high LVD and high BVD. Nine angiogenesis-related genes associated with the development of functional intratumoral lymhatics were identified. High expression of these genes, high LVD, and high BVD may be important biomarkers for poor outcome in cervix carcinoma.

## INTRODUCTION

Tumor growth in regional lymph nodes is an early sign of metastatic spread in cancer and is associated with poor prognosis in many histological types of carcinoma [1, 2]. Several features of the primary tumor associated with lymph node metastasis have been identified, including genomic alterations caused by defects in regulatory genes, the development of a pathophysiological microenvironment caused by abnormal angiogenesis, the evolvement of aberrant metabolic cell phenotypes caused by genetic and microenvironmental changes, and the presence of malignant cells with stem cell properties [3, 4]. Despite increased insights in recent years, the mechanisms leading to the development of lymph node metastases are far from fully understood. Even though the importance of angiogenesis in tumor growth was recognized by Folkman already in the early 1970s [5], the contribution of hemangiogenesis and lymphangiogenesis to the process of lymph node metastasis is still controversial [6].

There is strong evidence linking tumor hemangiogenesis to malignant progression and the development of metastatic disease [7]. Clinical studies have revealed that high blood vessel density (BVD) in the periphery of the primary tumor is associated with increased metastatic potential and poor survival in almost all types of cancer [8]. The evidence is particularly strong for breast carcinoma and some other tumor types that metastasize primarily to regional lymph nodes [9]. Tumor-induced lymphangiogenesis has also been linked to tumor aggressiveness and metastatic growth in several clinical investigations [2, 6]. Although intratumoral lymphatics have been detected in many cancer types, the importance of these lymphatics in lymph node metastasis is unclear. The lymphatics within human tumors appear to be functionally impaired [2, 6, 10], and studies of experimental tumors have suggested that tumors do not have functional intratumoral lymphatics [11, 12]. Nevertheless, studies involving tumors of several organs have suggested that high expression of the prolymphangiogenic factor vascular endothelial growth factor-C (VEGF-C) and high intratumoral or peritumoral lymph vessel density (LVD) are correlated with lymph node metastasis and poor prognosis [10, 13, 14].

Clinical investigations dealing with the role of angiogenesis in lymph node metastasis of carcinoma of the uterine cervix are sparse, and conflicting observations have been reported. Some investigations have revealed significant associations between high BVD in the primary tumor and lymph node metastasis [15–17], whereas others have suggested associations with tumor hypoxia rather than high BVD [18, 19]. Lymph node metastasis has also been reported to be correlated to high intratumoral LVD rather than high BVD [20], to high intratumoral LVD as well as high peritumoral LVD [21], and to high peritumoral LVD rather than high intratumoral LVD [22]. Furthermore, high density of leaky and tortuous blood vessels and impaired lymphatic drainage may cause elevated interstitial fluid pressure (IFP) in tumors [23], and high IFP in the primary tumor has been shown to be associated with lymph node involvement and poor survival rates in cervix cancer [24–26].

The importance of hemangiogenesis, lymphangiogenesis, and high IFP in lymph node metastasis of squamous cell carcinoma of the uterine cervix was examined in detail in this investigation. By performing experiments with patient-derived xenograft (PDX) models of cervix carcinoma, we have provided evidence that some tumor models can develop functional intratumoral lymphatics, and furthermore, that lymphangiogenesis induced by the primary tumor is an important determinant of lymph node metastasis in cervix cancer. The preclinical findings were supported by analysis of immunohistochemical preparations from tumor specimens derived from cervix cancer patients with locally advanced disease, revealing strong associations between intratumoral LVD, BVD, and the presence of pelvic lymph node metastases.

## RESULTS

### Aggressiveness and angiogenesis of PDX models and donor patients' tumors

Tumor aggressiveness and its relationship to hem- and lymphangiogenesis were investigated by studying two PDX models derived from highly aggressive cervix carcinomas (BK-12, LA-19), two PDX models derived from poorly aggressive cervix carcinomas (ED-15, HL-16), and tissue from the donor patients' tumors.

**Figure 1:**
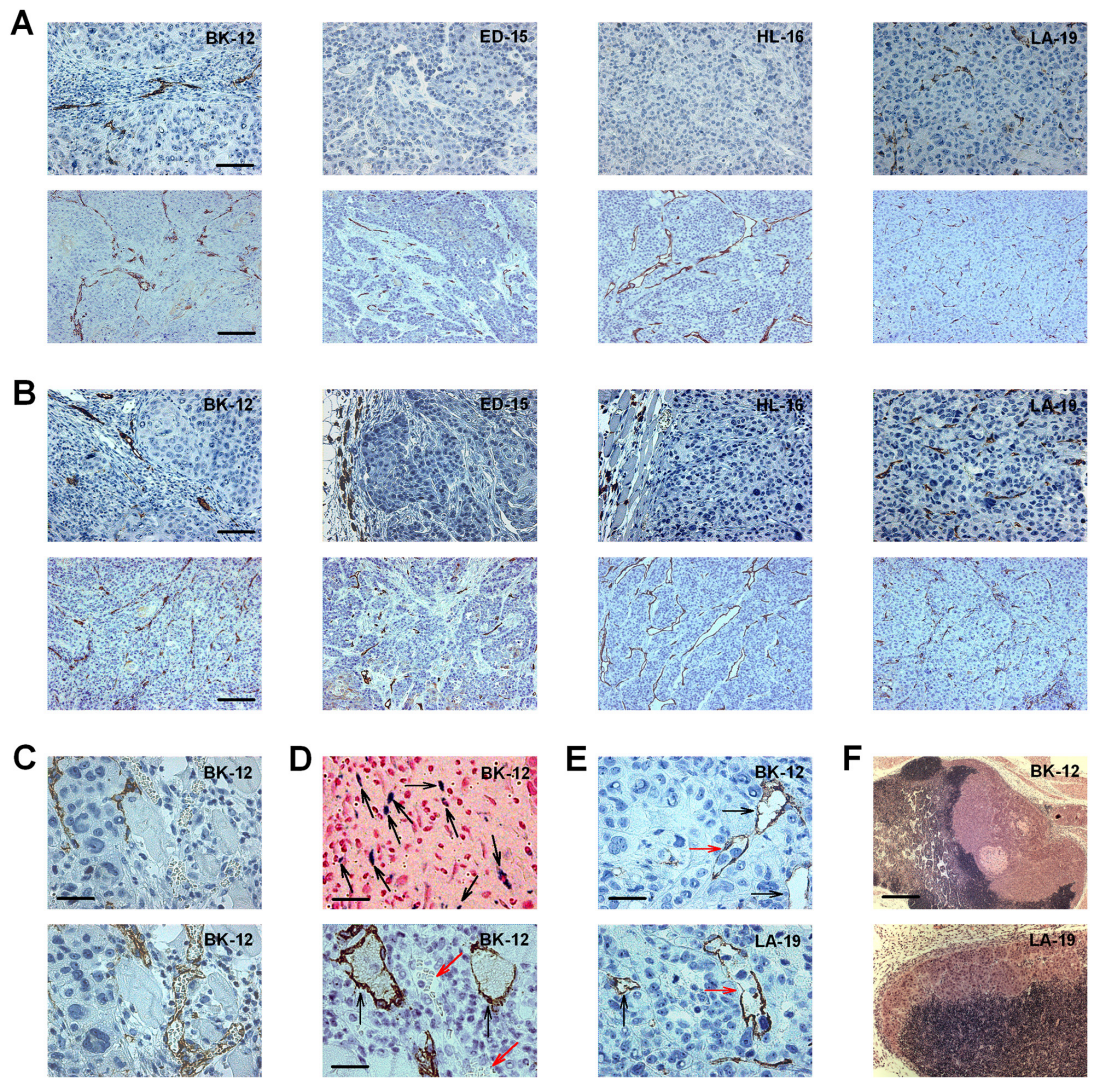
Histological appearance of BK-12, ED-15, HL-16, and LA-19 tumors **A.** Sections from donor patients' tumors stained with LYVE-1 (upper panels) or CD31 (lower panels). Bars, 100 μm (upper panels) or 200 μm (lower panels). **B.** Sections from patient-derived xenograft (PDX) tumors stained with LYVE-1 (upper panels) or CD31 (lower panels). Bars, 100 μm (upper panels) or 200 μm (lower panels). **C.** Adjacent sections from a BK-12 PDX tumor stained with LYVE-1 (upper panel) or CD31 (lower panel) showing that LYVE-1 and CD31 staining discriminated well between lymphatics and blood vessels. Bar, 25 μm. **D.** Images of a BK-12 PDX tumor showing functional intratumoral lymphatics. Upper panel, section stained with Prussian Blue showing many positively-stained low-diameter lymphatics (black arrows). Bar, 60 μm. Lower panel, section stained with LYVE-1 showing two positively-stained large-diameter ferritin-filled lymphatics (black arrows) and two unstained erythrocyte-containing blood vessels (red arrows). Bar, 30 μm. **E.** Sections from a BK-12 and an LA-19 PDX tumor stained with LYVE-1 showing intratumoral lymphatics with (red arrows) and without (black arrows) intraluminal tumor cells. Bar, 25 μm. **F.** Images showing metastatic growth in lymph nodes of mice bearing a BK-12 or an LA-19 PDX tumor. Bar, 650 μm.

**Figure 2:**
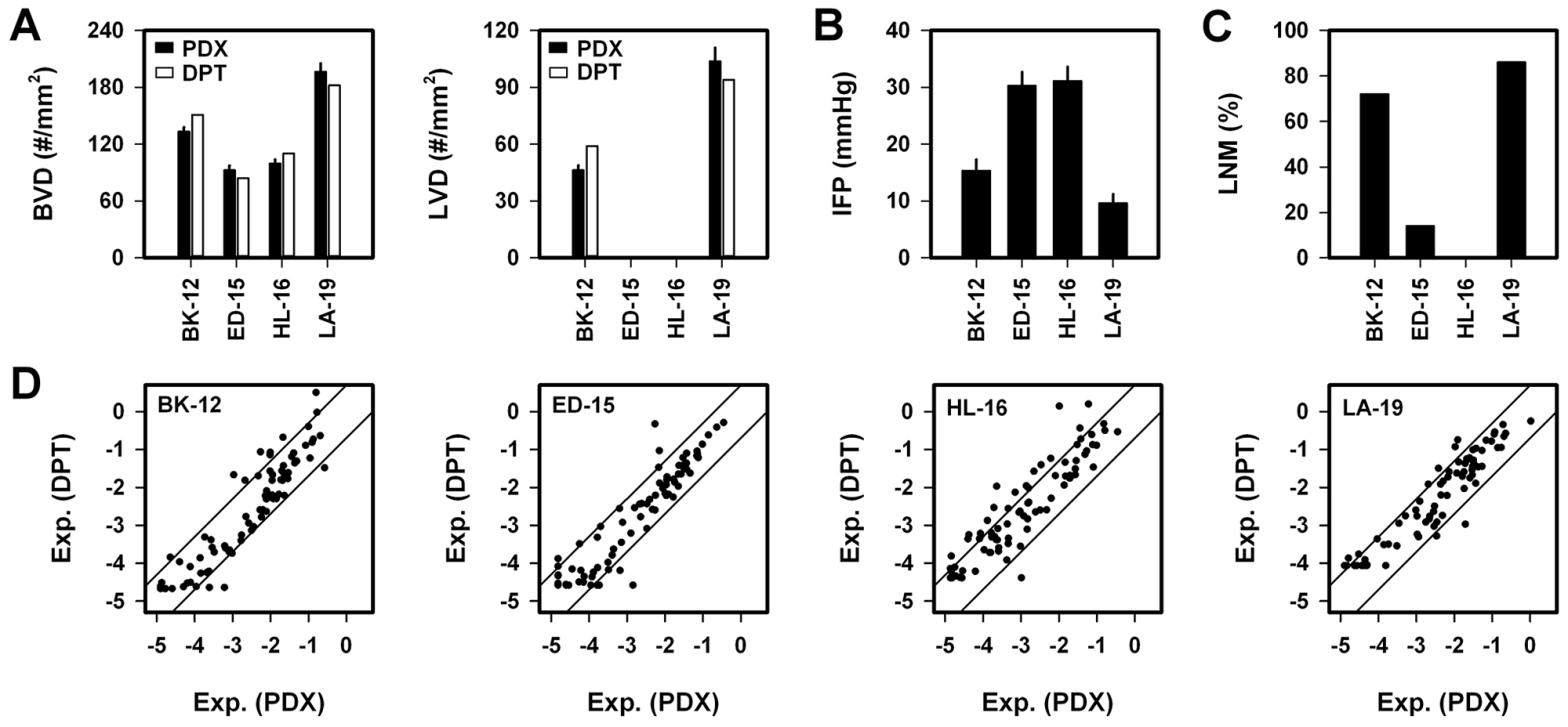
Blood vessel density (BVD), lymph vessel density (LVD), interstitial fluid pressure (IFP), lymph node metastasis (LNM), and expression of angiogenesis-related genes in BK-12, ED-15, HL-16, and LA-19 tumors **A.** BVD and LVD in donor patient's tumor (DPT) and patient-derived xenograft (PDX) model. Columns and bars, mean vessel counts based on six sections of each tumor (DPT) or mean ± SE of 20 tumors (PDX). **B.** Tumor IFP in PDX models. Columns and bars, mean ± SE of 20 (BK-12, ED-15, HL-16) or 10 (LA-19) tumors. **C.** Percentage of mice with LNM (n = 43–51 mice). **D.** Expression of angiogenesis-related genes in DPT *vs* PDX model. Axes, logarithmic scale from 10^−5^ to 10^0^. Symbols, mean values of individual genes based on three samples (DPT) or three tumors (PDX). Solid lines, 5-fold difference in expression between DPT and PDX.

Tumor vascularization was studied qualitatively (Figure 1) and quantitatively (Figure 2) by examining histological preparations. Two of the donor patients' tumors (BK-12 and LA-19) showed intratumoral lymphatics, whereas the other two (ED-15 and HL-16) did not (Figure 1A). The blood vessels of the BK-12, ED-15, and HL-16 tumors were located primarily within stromal connective tissue, whereas the LA-19 tumor frequently showed blood vessels also in the parenchyma (Figure 1A). Similar to the donor patients' tumors, the BK-12 and LA-19 PDX models showed both intratumoral and peritumoral lymphatics, whereas the ED-15 and HL-16 models showed lymphatics only in peritumoral tissue (Figure 1B). Moreover, the blood vessel staining pattern of the PDX models was similar to that of the donor patients' tumors (Figure 1B).

Adjacent histological sections were subjected to immunohistochemistry to investigate whether vessels showed receptors for both CD31 and LYVE-1. Less than 2 % of the vessels stained positive for both CD31 and LYVE-1, suggesting that CD31 and LYVE-1 immunohistochemistry discriminated well between blood vessels and lymphatics in BK-12 and LA-19 tumors (Figure 1C).

To investigate whether BK-12 and LA-19 tumors showed functional lymphatics, ferritin was injected within tumors before histological sections were prepared and stained for ferritin and LYVE-1. LYVE-1-positive vessels with intraluminal ferritin were seen frequently in both the periphery and central regions of BK-12 and LA-19 tumors (Figure 1D), suggesting the presence of functional intratumoral lymphatics in both tumor models.

Interestingly, single tumor cells or clusters of tumor cells located within the lumen of intratumoral lymphatics were seen in histological sections prepared from BK-12 and LA-19 tumors (Figure 1E), and mice bearing BK-12 or LA-19 tumors frequently showed macroscopic tumor growth in lymph nodes (Figure 1F).

Quantitative studies revealed that the PDX models mirrored the angiogenic properties of the donor patients' tumors and that BVD was higher in BK-12 and LA-19 tumors than in ED-15 and HL-16 tumors (*P* = 0.0008) and that LVD was higher in LA-19 tumors than in BK-12 tumors (*P* < 0.0001; Figure 2A). Moreover, IFP was lower in BK-12 and LA-19 tumors than in ED-15 and HL-16 tumors (*P* < 0.0001; Figure 2B). The BK-12 and LA-19 models were highly metastatic, whereas the ED-15 and HL-16 models were poorly and non-metastatic, respectively (Figure 2C). Thus, the metastatic propensity of the tumor models mirrored the aggressiveness of the donor patients' tumors and was associated with their ability to develop functional intratumoral lymphatics. Compared with the poorly/non-metastatic models, the highly metastatic models with functional intratumoral lymphatics showed low IFP, high LVD, and high BVD.

The expression of angiogenesis-related genes in the donor patients' tumors and the PDX models was studied by quantitative PCR (Supplementary Table S1). These studies revealed that the expression in the PDX models in general reflected that in the donor patients' tumors (Figure 2D). However, six genes showed >2-fold higher expression in all donor patients' tumors than in the corresponding PDX model, whereas no gene showed >2-fold higher expression in all PDX models than in the corresponding donor patient's tumor (Supplementary Table S2). Moreover, the expression levels differed among the PDX models and were generally higher in the BK-12 and LA-19 models than in the ED-15 and HL-16 models (Supplementary Figure S1). Fifteen genes showed >2-fold higher expression in the BK-12 and LA-19 models than in the ED-15 and HL-16 models, whereas only one gene showed >2-fold higher expression in the ED-15 and HL-16 models than in the BK-12 and LA-19 models (Supplementary Table S3).

### IFP, angiogenesis, and lymph node metastasis of individual tumors of the PDX models

**Figure 3:**
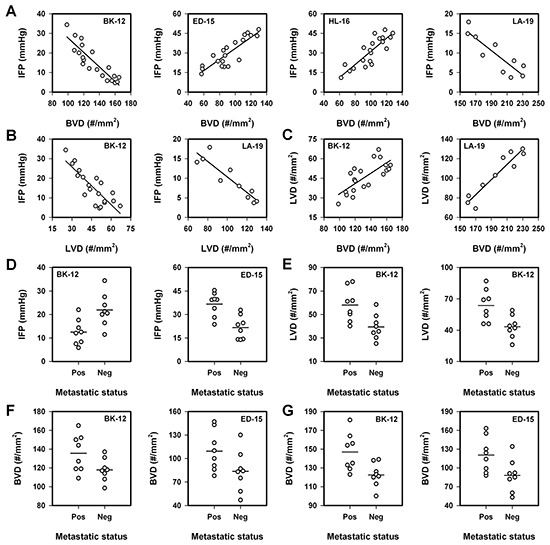
Interstitial fluid pressure (IFP), blood vessel density (BVD), lymph vessel density (LVD), and lymph node metastasis in BK-12, ED-15, HL-16, and LA-19 tumors **A.** IFP *vs* BVD. **B.** IFP *vs* LVD. **C.** LVD *vs* BVD. **A–C.** Symbols, individual tumors. Curves, linear regression lines. **D.** IFP in metastatic and non-metastatic tumors. **E.** LVD in metastatic and non-metastatic tumors. Left panel, whole tumor cross-sections. Right panel, periphery of tumor cross-sections. **F.** BVD in whole cross-sections of metastatic and non-metastatic tumors. **G.** BVD in the periphery of cross-sections of metastatic and non-metastatic tumors. D–G. Symbols, individual tumors. Horizontal lines, mean values.

Associations between IFP and angiogenesis were searched for in each PDX model by subjecting tumor-bearing mice to measurement of tumor IFP before the tumors were resected and immunostained for assessment of BVD and LVD. IFP, BVD, and LVD differed among individual tumors of the same model, and significant correlations were found between IFP and BVD (Figure 3A) and IFP and LVD (Figure 3B). IFP increased with increasing BVD in ED-15 (*P* < 0.0001) and HL-16 (*P* < 0.0001) tumors and decreased with increasing BVD in BK-12 (*P* < 0.0001) and LA-19 (*P* = 0.0009) tumors. These correlations were thus different for the models with and the models without functional intratumoral lymphatics. Moreover, IFP also decreased with increasing LVD (*P* < 0.0001, BK-12; *P* = 0.0003, LA-19), and there were positive correlations between LVD and BVD in the BK-12 (*P* < 0.0001) and LA-19 (*P* < 0.0001) models (Figure 3C).

To search for associations between lymph node metastasis, IFP, BVD, and LVD, eight metastatic and eight non-metastatic tumors of the BK-12 and ED-15 models were studied. In the BK-12 model, the metastatic tumors showed lower IFP than the non-metastatic tumors (*P* = 0.0091), whereas the metastatic tumors showed higher IFP than the non-metastatic tumors in the ED-15 model (*P* = 0.013, Figure 3D). The LVD was higher in the metastatic than in the non-metastatic BK-12 tumors, independent of whether the total areas (*P* = 0.0098) or only the peripheral regions (*P* = 0.0075) of tumor cross-sections were analyzed (Figure 3E). Moreover, the metastatic tumors showed higher BVD than the non-metastatic tumors in both models (Figure 3F and G). The differences were of borderline significance when whole tumor cross-sections were scored (*P* = 0.049, BK-12; *P* = 0.066, ED-15; Figure 3F) and highly significant when only the tumor periphery was considered (*P* = 0.011, BK-12; *P* = 0.029, ED-15; Figure 3G).

### Differences between tumors of the BK-12 model in early and late passages

**Figure 4:**
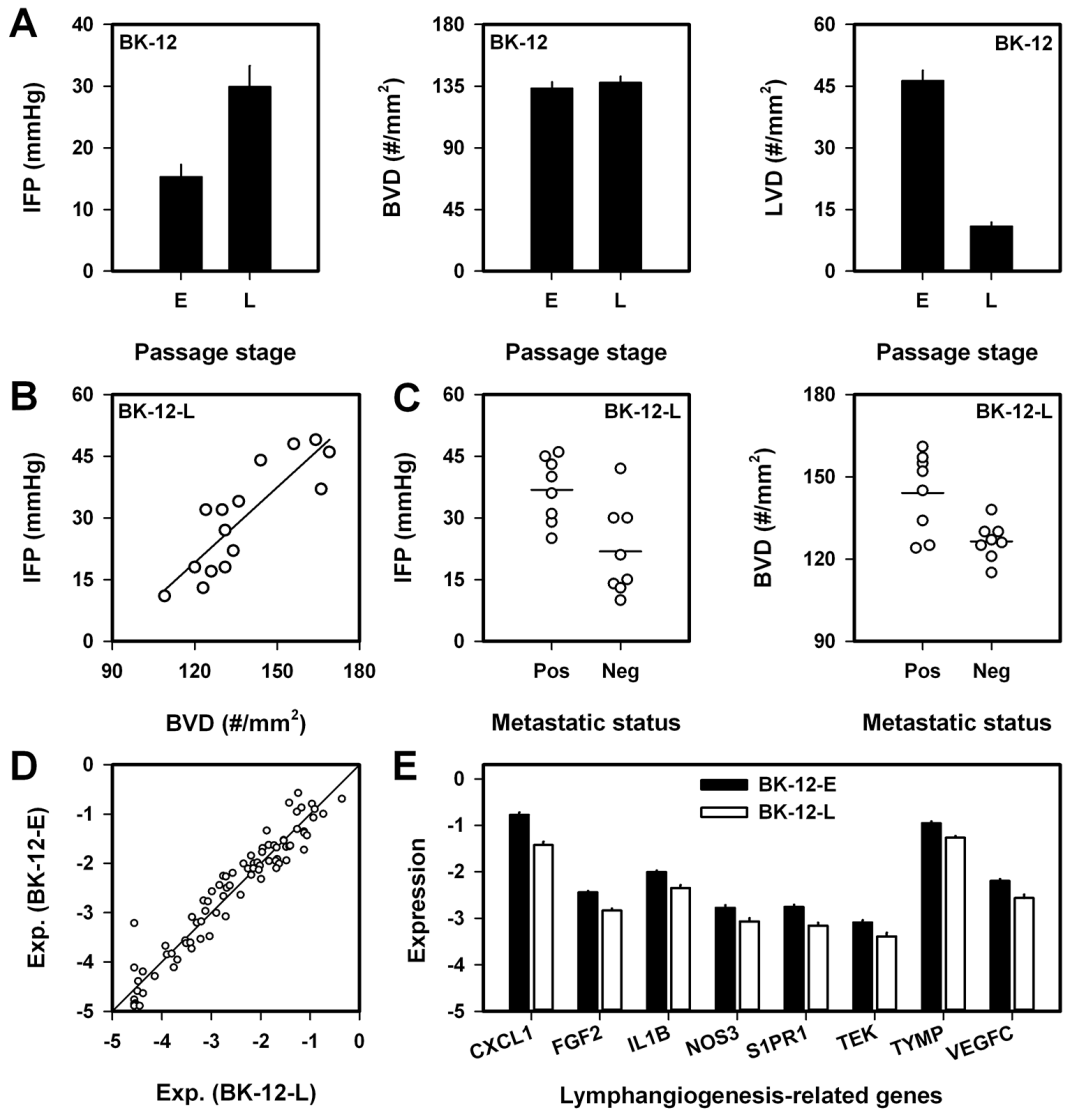
Interstitial fluid pressure (IFP), blood vessel density (BVD), lymph vessel density (LVD), lymph node metastasis, and expression of angiogenesis-related genes in BK-12 tumors in early (BK-12-E) and late (BK-12-L) passages (passages 4–5 vs 15–20) **A.** IFP, BVD, and LVD in BK-12 tumors in early (E) and late (L) passages. Columns and bars, mean ± SE of 15 tumors. **B.** IFP *vs* BVD in BK-12-L tumors. Symbols, individual tumors. Curve, linear regression line. **C.** IFP and BVD in metastatic and non-metastatic BK-12-L tumors. Symbols, individual tumors. Horizontal lines, mean values. **D.** Expression of angiogenesis-related genes in BK-12-E *vs* BK-12-L tumors. Axes, logarithmic scale from 10^−5^ to 10^0^. Symbols, mean values of individual genes based on three tumors of each category. Solid line, no difference in expression between BK-12-E and BK-12-L tumors. **E.** Expression of lymphangiogenesis-related genes in BK-12-E and BK-12-L tumors. Ordinate, logarithmic scale from 10^−5^ to 10^0^. Columns and bars, mean ± SE of 3 tumors.

The growth rate of BK-12 tumors increased gradually during serial transplantation, and by comparing tumors in late passages (BK-12-L, passages 15-20) with tumors in early passages (BK-12-E, passages 4-5), significant differences were detected. BK-12-L tumors showed higher IFP (*P* = 0.0009) and lower LVD (*P* < 0.0001) than BK-12-E tumors, whereas BVD was similar (Figure 4A). Furthermore, IFP increased with increasing BVD in BK-12-L tumors (*P* < 0.0001, Figure 4B), in contrast to what was observed in BK-12-E tumors (Figure 3A). Any correlation between LVD and IFP or BVD could not be detected in BK-12-L tumors, possibly because LVD was too low. Moreover, functional lymphatics could not be detected in BK-12-L tumors by using the ferritin assay.

Also BK-12-L tumors developed lymph node metastases frequently, and to investigate whether lymph node metastasis was associated with IFP and/or BVD, an experiment similar to that described above for BK-12-E tumors was carried out. Eight metastatic and eight non-metastatic tumors were examined, and as opposed to metastatic BK-12-E tumors (Figure 3D), metastatic BK-12-L tumors had higher IFP than their non-metastatic counterparts (*P* = 0.0077, Figure 4C). Furthermore, BVD was higher in metastatic than in non-metastatic BK-12-L tumors (*P* = 0.0081, Figure 4C), similar to what was observed in BK-12-E tumors (Figure 3F).

Large differences between BK-12-E and BK-12-L tumors in the expression of angiogenesis-related genes could not be detected by quantitative PCR (Figure 4D). However, many genes showed a >2-fold difference in the expression level, and this difference was statistically significant for 24 genes (Supplementary Table S4). Eight of these genes have been shown to play an important role in lymphangiogenesis, and the expression level of all these genes was higher in BK-12-E than in BK-12-L tumors (Figure 4E).

Of the 84 genes included in the PCR angiogenesis array, 9 genes were found to show significantly higher expression in the BK-12 and LA-19 models than in the ED-15 and HL-16 models as well as significantly higher expression in BK-12-E than in BK-12-L tumors (Table 1). Table 1 thus lists the genes that were found to be associated with the presence of functional intratumoral lymphatics in PDXs, and noteworthy, this list contains the well established lymph angiogenic factor VEGF-C, but not VEGF-A, VEGF-B, or VEGF-D (FIGF, c-fos induced growth factor). No angiogenesis-related gene showed significantly lower expression in tumors with functional intratumoral lymphatics than in those without functional lymphatics within the tumor mass.

**Table 1:** Angiogenesis-related genes associated with the presence of functional intratumoral lymphatics in PDX models of squamous cell carcinoma of the uterine cervix

Gene refseq^*^	Symbol	Name^**^
NM_001565	CXCL10	Chemokine (C-X-C motif) ligand 10
NM_001993	F3	Coagulation factor III (thromboplastin, tissue factor)
NM_002026	FN1	Fibronectin 1
NM_000618	IGF1	Insulin-like growth factor 1 (somatomedin C)
NM_000576	IL1B	Interleukin 1, beta
NM_001400	S1PR1	Sphingosine-1-phosphate receptor 1
NM_000459	TEK	TEK tyrosine kinase, endothelial (Tie-2)
NM_003254	TIMP1	TIMP metallopeptidase inhibitor 1
NM_005429	VEGFC	Vascular endothelial growth factor C

* NCBI reference sequence database.

** Genes showing >2-fold higher expression in the BK-12 and LA-19 models than in the ED-15 and HL-16 models as well as >2-fold higher expression in BK-12-E tumors than in BK-12-L tumors. *P* < 0.05 for all genes listed in the table.

### BVD, LVD, and lymph node metastasis in human cervix carcinoma

**Figure 5:**
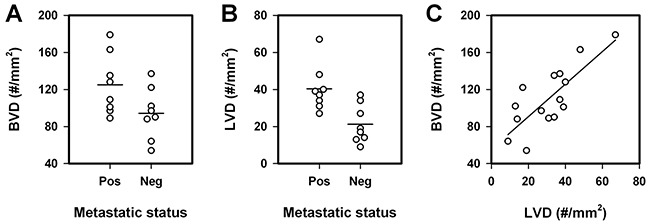
Blood vessel density (BVD), lymph vessel density (LVD), and lymph node metastasis in human cervix carcinoma **A.** BVD of metastatic and non-metastatic tumors. **B.** LVD of metastatic and non-metastatic tumors. **C.** BVD *vs* LVD. Symbols, individual patients' primary tumor. Horizontal lines, mean values. Curve, linear regression line.

To investigate whether our preclinical observations are consistent with human cervix cancer data, primary tumor BVD and LVD were measured in eight patients with extensive lymph node involvement and eight patients without detectable lymph node metastasis. Two biopsies and three sections of each biopsy were analyzed for each tumor. The lymph node positive patients showed higher BVD (*P* = 0.029, Figure 5A) and higher LVD (*P* = 0.0048, Figure 5B) than the lymph node negative patients, and moreover, there was a significant correlation between BVD and LVD (*P* = 0.0003, Figure 5C), in accordance with the preclinical data.

## DISCUSSION

Immunohistochemical investigations have revealed that intratumoral lymphatics may be present in many cancer types [27, 28]; however, there is an open question whether these vessels are functional [29]. Studies of experimental tumors have suggested that intratumoral lymphatics may be nonfunctional [30]. Thus, transplantation of VEGF-C overexpressing tumor cells to mice has initiated tumors showing peritumoral lymphatic hyperplasia and increased lymph drainage, but no functional intratumoral lymphatics [11, 31]. These studies were carried out with tumors initiated from cell lines established in culture, and the lack of functional intratumoral lymphatics has been attributed to vessel compression caused by rapid tumor growth [12].

By using PDX models of cervix carcinoma reflecting the biology of the donor patients' tumors, this study provides evidence that some tumors may develop functional intratumoral lymphatics, whereas other tumors may show functional lymphatics only in peritumoral regions. In contrast to ED-15 and HL-16 tumors, BK-12 and LA-19 tumors developed high densities of intratumoral lymphatics, and the ferritin experiments reported here suggest that some of these lymphatics were functional. Furthermore, BK-12 and LA-19 tumors had lower IFP than ED-15 and HL-16 tumors. However, although functional lymphatics were seen within BK-12 and LA-19 tumors, these tumors still showed moderately elevated IFP values, suggesting that the intratumoral lymphatic network had impaired functionality compared with the peritumoral lymphatic network.

It has been suggested that PDX models of cancer should be transplanted to orthotopic sites to allow the tumor cells to interact with the most relevant organ microenvironment [32, 33]. The tumors examined in this study were transplanted intramuscularly rather than orthotopically because orthotopic transplantation of cervix carcinomas is technically challenging and thus not well suited for large scale studies. It should be noted that studies of cell line-derived cervix carcinoma xenografts have revealed that the IFP is higher in intramuscular tumors than in tumors growing in the cervix [34].

The two PDX models with functional intratumoral lymphatics showed higher BVD and higher expression of angiogenesis-related genes than the other two models. The intertumor heterogeneity of each PDX model was substantial, and studies of individual BK-12 and LA-19 tumors revealed significant correlations between LVD and BVD. Furthermore, a significant correlation was found between LVD and BVD in tumor biopsies from cervix cancer patients. Taken together, these observations suggest that lymphangiogenesis is associated with hemangiogenesis in cervix carcinoma.

Significant correlations between IFP and vessel density were detected by examining individual tumors of each PDX model, and these correlations were different for the models that showed functional intratumoral lymphatics and those that did not. Thus, IFP decreased with increasing BVD and LVD in BK-12 and LA-19 tumors and increased with increasing BVD in ED-15 and HL-16 tumors. The ED-15 and HL-16 data are consistent with the prevailing view that high IFP in tumors is a consequence of lack of functional intratumoral lymphatics and high resistance to blood flow, and furthermore, that high BVD is an important cause of high geometric resistance to blood flow in tumors [23, 35]. The BK-12 and LA-19 data are consistent with the finding that these tumors developed functional lymphatics within the tumor mass, and they suggest that the functionality of the intratumoral lymphatic network increased with increasing LVD. Functional intratumoral lymphatics may facilitate the transport of interstitial fluid from tumors into the circulatory system and may thus prevent the development of a high tumor IFP.

The PDX models with functional intratumoral lymphatics showed a substantially higher incidence of lymph node metastases than the PDX models without intratumoral lymphatics. The distances between tumor cells and lymphatics are short in tumors having lymphatics within the tumor mass, and functional intratumoral lymphatics could promote lymph node metastasis by facilitating tumor cell intravasation and transport to the draining lymph nodes. However, even though tumor cells were observed frequently within the lumen of intratumoral lymphatics in BK-12 and LA-19 tumors, the tumor cells may not necessarily have used this escape route when metastasizing. Tumor-secreted cytokines may promote metastatic growth in lymph nodes by stimulating the development of a premetastatic lymphovascular niche prior to the arrival of tumor cells [6, 10, 13, 14], and functional intratumoral lymphatics could promote lymph node metastasis by facilitating the transport of cytokines and the development of a premetastatic niche. Early development of a premetastatic lymphovascular niche may have contributed significantly to the high metastatic propensity of BK-12 and LA-19 tumors because the host mice of these tumors showed greatly enlarged lymph nodes in tumor draining regions several days before metastatic growth could be detected by histological examinations.

In addition to having high LVD and functional intratumoral lymphatics, the BK-12 and LA-19 models showed higher BVD than the ED-15 and HL-16 models. Although it is possible that tumor cells circulating in the blood may home to lymph nodes, this route of lymph node metastasis is considered to be extraordinarily unusual [2]. BK-12 and LA-19 tumors developed metastases mainly in the sentinel and downstream lymph nodes, suggesting that the metastatic cells of these tumors escaped through intratumoral and peritumoral lymphatics rather than through blood vessels.

LVD decreased and IFP increased during serial transplantation of BK-12 tumors, and functional intratumoral lymphatics could not be detected in BK-12-L tumors with the ferritin assay. Studies of individual BK-12-L tumors revealed significant correlations between lymph node metastasis, IFP, and BVD, and these correlations were different from those in BK-12-E tumors but similar to those in ED-15 tumors. Consequently, these data add further evidence to our suggestion that tumors with and without functional intratumoral lymphatics may have substantially different biological properties.

Cervix cancer patients with lymph node metastases have poor prognosis, and many investigators have searched for correlations between lymph node metastasis and biological properties of the primary tumor in attempts to identify biomarkers associated with treatment failure. Our preclinical observations suggest that high BVD and LVD in the tumor periphery may be important biomarkers for high metastatic propensity in cervix carcinoma, independent of whether the tumors have functional intratumoral lymphatics or not. This suggestion is supported by the clinical observations reported here, and furthermore, it is in accordance with patient data from a large number of other cancer types having shown that high BVD in the tumor periphery is associated with high incidence of lymph node metastases and poor survival rates [8, 36].

Several investigations have provided evidence that high IFP in the primary tumor may be a powerful biomarker for poor outcome in cervix carcinoma. Studies of patients with locally advanced cervix cancer have suggested that high IFP is linked to elevated incidence of lymph node metastases, pelvic recurrence after radiation therapy, and poor disease-free and overall survival rates [24–26]. Moreover, studies of cell line-derived cervix carcinoma xenografts have revealed that the development of lymph node metastasis may be associated with high IFP in the primary tumor [37]. There is an apparent discrepancy between these data and the PDX data reported here. It is possible that only a small fraction of the patients with locally advanced cervix cancer have primary tumors with functional intratumoral lymphatics and that the BK-12 and LA-19 models represent this subpopulation.

Cervix cancer patients with functional lymphatics within central tumor regions may have particularly poor prognosis. Nine angiogenesis-related genes were identified that showed significantly elevated expression in all tumor models with functional intratumoral lymphatics. These genes are involved in different signaling pathways [10], suggesting that the mechanisms leading to the development of functional lymphatics within tumors may be multiple and complex. It is possible that high expression of these genes may be important biomarkers for poor treatment outcome in cervix carcinoma, a possibility that merits to be investigated thoroughly in prospective clinical studies.

Interestingly, a recent study showed that RAS association domain family 8 (RASSF8) downregulation increases VEGF-C expression, promotes lymphangiogenesis and lymph node metastasis, and is associated with poor patient survival in esophageal squamous cell carcinoma [38]. Based on these observations, the authors suggested that downregulated RASSF8 may be a biomarker for poor outcome and a therapeutic target in squamous cell carcinoma.

In summary, our study has revealed that some PDX models of squamous cell carcinoma of the uterine cervix may develop functional intratumoral lymphatics during growth. Tumors with functional intratumoral lymphatics have high LVD, high BVD, high expression of angiogenesis-related genes, low IFP, and show high incidence of lymph node metastases. Precision medicine may benefit from assays predicting the presence of functional lymphatics within tumors, and this study has detected nine angiogenesis-related genes that are potentially useful biomarkers for functional intratumoral lymphatics in cervix carcinoma.

## MATERIALS AND METHODS

### PDX models

Adult (8–12 weeks of age) female BALB/c *nu*/*nu* mice were used as host animals for xenografted tumors. Four PDX models (BK-12, ED-15, HL-16, and LA-19) of squamous cell carcinoma of the uterine cervix, established from patients with FIGO stage IIB disease prior to treatment, were included in the study. The patients were given radiation therapy with curative intent as combined external irradiation and endocavitary brachytherapy, as described in detail elsewhere [39]. The donor patients of the BK-12 and LA-19 models had developed several pathological lymph nodes at presentation and died from their diseases within 3 years after the initial diagnosis, whereas the donors of the ED-15 and HL-16 models presented without detectable lymph node involvement and responded well to treatment. The BK-12 and LA-19 models were thus derived from patients with particularly aggressive diseases, in contrast to the ED-15 and HL-16 models.

We have established a frozen stock of cell suspensions from xenografted tumors in passage 2. Unless otherwise stated, the experiments reported here were carried out with intramuscular tumors in passage 4 or 5. These tumors were initiated in the right quadriceps femoris of mice by inoculating aliquots of 5 × 10^5^ cells derived from intramuscular tumors initiated from the frozen stock.

### Blood and lymph vessel density in PDXs

Histological sections were prepared by standard procedures and immunostained for blood vessels or lymphatics as described previously [40]. CD31 and LYVE-1 were used as markers of blood and lymph vessel endothelial cells, respectively. An anti-mouse CD31 rabbit polyclonal antibody (Abcam, Cambridge, UK) or an anti-mouse LYVE-1 rabbit polyclonal antibody (Abcam) was used as primary antibody. Quantitative studies were carried out on preparations cut through the central regions of tumors, and three sections of each staining were analyzed for each tumor. Microvessels were defined and scored manually as described elsewhere [36, 41]. Peripheral vessel density was determined by counting vessels located within a 1-mm-thick rim in the tumor periphery [40]. BVD and LVD were scored as number of vessels per mm^2^ of viable tumor tissue.

### Quantitative PCR

The RT^2^ Profiler PCR Array Human Angiogenesis (PAHS-024Z) from SABiosciences (Frederick, MD, USA) was used for expression profiling of angiogenesis-related genes. Total RNA was isolated from tumor tissue stabilized in RNA*later* RNA Stabilization Reagent (Qiagen, Hilden, Germany). RNA isolation, cDNA synthesis, and real-time PCR were carried out as described earlier [42]. Fold difference in gene expression was calculated by using the ΔΔC_T_-method [43]. A C_T_-value of 35 (15 cycles above the positive PCR control) was set as detection limit, and hence, all C_T_-values above 35 were set to 35. The arrays included 5 housekeeping genes [β-actin (ACTB), β-2-microglobulin (B2M), glyceraldehyde-3-phosphate dehydrogenase (GAPDH), hypoxanthine phosphoribosyltransferase 1 (HPRT1), ribosomal protein lateral stalk subunit P0 (RPLP0)], and each C_T_-value of a sample was normalized to the mean C_T_-value of these genes (ΔC_T_ = C_T_^gene of interest^ - C_T_^mean of housekeeping genes^). Normalized gene expression levels were calculated from three tumors (PDX models) or three independent tissue samples from a single biopsy (donor patients' tumors) as 2^−mean ΔCT^. The genes included in the PCR array are listed in Supplementary Table S1.

### Lymph node metastasis

Tumor-bearing mice were euthanized and examined for external lymph node metastases in the inguinal, axillary, interscapular, and submandibular regions, and internal lymph node metastases in the abdomen and mediastinum [44]. Metastatic growth in enlarged lymph nodes was confirmed by histological examination.

### Functionality of lymphatics

Ferritin lymphangiography was used to study the functionality of intratumoral lymphatics. Approximately 5 μl of ferritin solution (~100 mg/ml) was injected slowly into the center of tumors, and 45 min after the administration, the tumors with surrounding normal tissue were excised, fixed in phosphate-buffered 4% paraformaldehyde, and embedded in paraffin. Sections (4-μm thick) were cut, and the iron III component of ferritin was revealed by staining with Prussian Blue, using a 5% potassium ferrocyanide and 10% HCl solution [31]. Immunostaining with LYVE-1 was used to confirm that ferritin-positive structures were lymphatics.

### Interstitial fluid pressure

IFP was measured in the center of tumors with a Millar SPC 320 catheter equipped with a 2F Mikro-Tip transducer (Millar Instruments, Houston, TX). The catheter was connected to a computer via a Millar TC-510 control unit and a preamplifier, and data acquisition was carried out by using the LabVIEW software. By measuring IFP in the same tumors twice, we have shown that this method produces highly reproducible IFP values [45].

### Human cervix carcinoma

Associations between MVD, LVD, and lymph node metastasis were studied in 16 patients with FIGO stage IIB squamous cell carcinoma of the uterine cervix. Eight patients with metastases in at least three pelvic lymph nodes and 8 patients without detectable lymph node involvement were selected for the study. The patient selection was based on examination of *T*_2_-weighted fast spin echo MR images of the pelvis, covering the internal, external, and lower common iliac chains. A lymph node was considered to be metastasis-positive when its shortest diameter was longer than 1.0 cm and metastasis-negative when its longest diameter was shorter than 1.0 cm.

Tumor biopsies were removed from the primary tumor of the patients with a curette, and histological sections were prepared and immunostained for blood vessels and lymphatics by using standard methods. An anti-human CD31 rabbit polyclonal antibody (Abcam) or an anti-human LYVE-1 rabbit polyclonal antibody (Abcam) was used as primary antibody. Two biopsies from different tumor regions and three sections of each biopsy were analyzed for each patient. Microvessels were defined and scored manually as described elsewhere [36, 41].

### Ethics

The excision of tumor tissue for establishing PDX models of cervix carcinoma was approved by the Institutional Ethics Committee. Informed consent was obtained from the donor patients. The animal experiments were approved by the Institutional Committee on Research Animal Care and were performed in accordance with the Interdisciplinary Principles and Guidelines for the Use of Animals in Research, Marketing, and Education (New York Academy of Sciences, New York, NY, USA) and the EU Directive 2010/63/EU for animal experiments (http://ec.europa.eu/environment/chemicals/lab_animals/legislation_en.htm). The studies investigating relationships between BVD, LVD, and metastatic status in human cervix cancer were approved by the regional committee of medical research ethics and were conducted according to the Declaration of Helsinki. Informed consent was obtained from each patient.

### Statistical analysis

Data are shown as mean ± standard error unless otherwise stated. The Pearson product moment correlation test was used to search for correlations between parameters. Curves were fitted to data by linear regression analysis. Comparisons of data were carried out by using the Student *t* test (single comparisons) or by one-way ANOVA followed by the Bonferroni's test (multiple comparisons) when the data complied with the conditions of normality and equal variance. Under other conditions, comparisons were carried out by nonparametric analysis using the Mann-Whitney rank-sum test (single comparisons) or by Kruskal-Wallis ANOVA on ranks followed by the Dunn's test (multiple comparisons). The Kolmogorov-Smirnov method and the Levene's method were used to test for normality and equal variance, respectively. Probability values of *P* < 0.05, determined from two-sided tests, were considered significant. The statistical analysis was carried out with SigmaStat statistical software.

## SUPPLEMENTARY MATERIALS FIGURE AND TABLES










