# Supplementary material for: Functional intratumoral lymphatics in patient-derived xenograft models of squamous cell carcinoma of the uterine cervix: implications for lymph node metastasis
Source: Oncotarget. 2016 Jul 29;7(35):56986–97. doi: 10.18632/oncotarget.10931 (PMC5302967; doi:10.18632/oncotarget.10931)
Supplement: Supplementary file 1 [file oncotarget-07-56986-s001.pdf]

Functional intratumoral lymphatics in patient-derived xenograft models of squamous cell carcinoma of the uterine cervix: implications for lymph node metastasis

SUPPLEMENTARY FIGURE AND TABLES

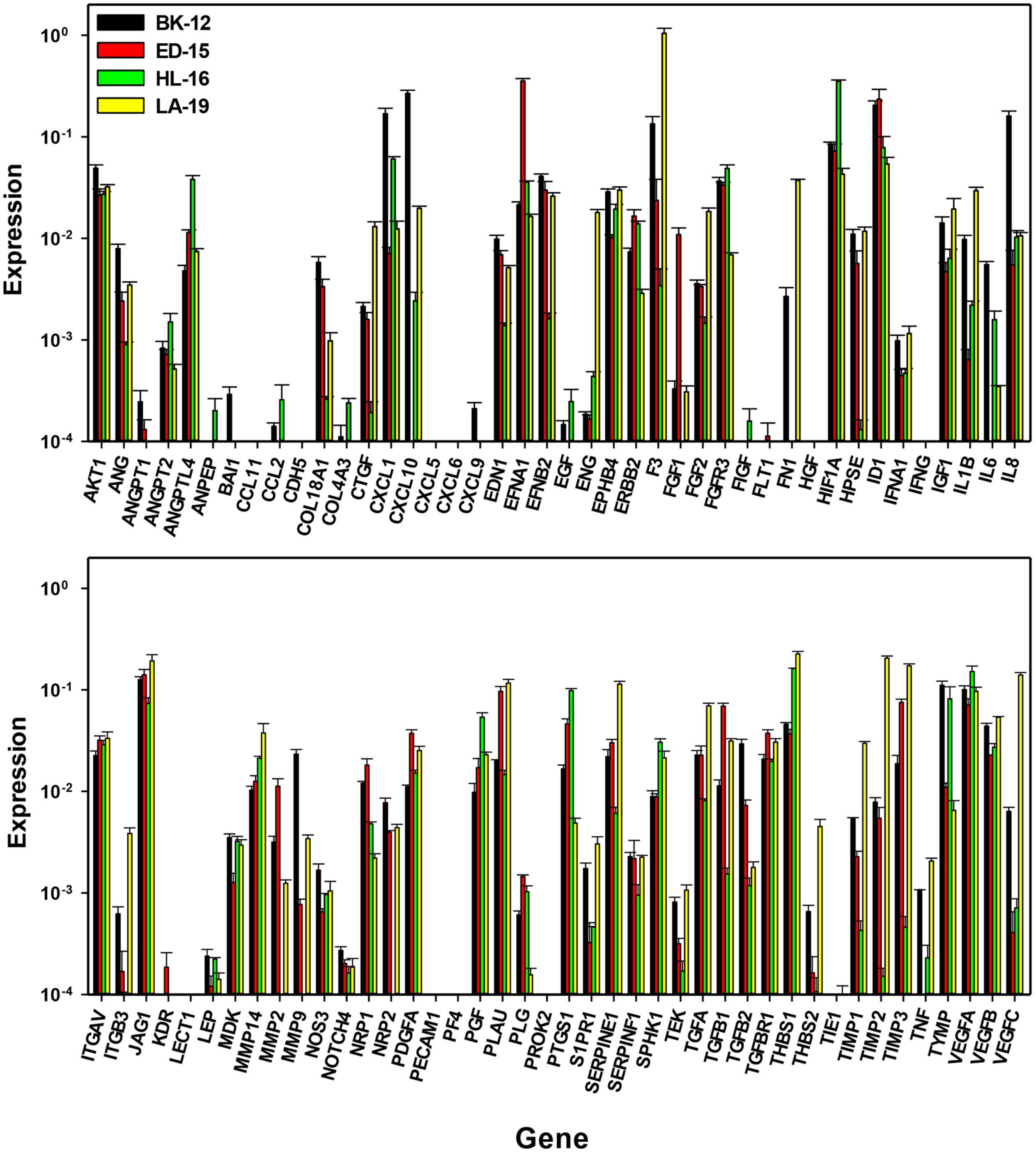

Supplementary Figure S1: Relative expression levels of angiogenesis-related genes in the BK-12, ED-15, HL-16, and LA-19 PDX models. Columns and bars, mean ± standard deviation (n = 3).

Supplementary Table S1: Angiogenesis-related genes included in the PCR array*.

See Supplementary File 1

Supplementary Table S2: Fold difference in gene expression between donor patient's tumor (DPT) and patient-derived xenograft (PDX) model

See Supplementary File 2

Supplementary Table S3: Fold difference in gene expression between highly metastatic (BK-12, LA-19) and poorly/ non-metastatic (ED-15, HL-16) PDX models.

See Supplementary File 3

Supplementary Table S4: Genes with expression levels that differed between BK-12-E and BK-12-L tumors by a factor of at least 2*.

See Supplementary File 4
