# Supplementary material for: Functional intratumoral lymphatics in patient-derived xenograft models of squamous cell carcinoma of the uterine cervix: implications for lymph node metastasis
Source: Oncotarget. 2016 Jul 29;7(35):56986–97. doi: 10.18632/oncotarget.10931 (PMC5302967; doi:10.18632/oncotarget.10931)
Supplement: Supplementary file 2 [file oncotarget-07-56986-s002.docx]

**Supplementary Table S1.** Angiogenesis-related genes included in the PCR array*

| **Gene Refseq**** | **Symbol** | **Name** |
| --- | --- | --- |
| NM_005163 | AKT1 | V-akt murine thymoma viral oncogene homolog 1 |
| NM_001145 | ANG | Angiogenin. ribonuclease. RNase A family. 5 |
| NM_001146 | ANGPT1 | Angiopoietin 1 |
| NM_001147 | ANGPT2 | Angiopoietin 2 |
| NM_001039667 | ANGPTL4 | Angiopoietin-like 4 |
| NM_001150 | ANPEP | Alanyl (membrane) aminopeptidase |
| NM_001702 | BAI1 | Brain-specific angiogenesis inhibitor 1 |
| NM_002986 | CCL11 | Chemokine (C-C motif) ligand 11 |
| NM_002982 | CCL2 | Chemokine (C-C motif) ligand 2 |
| NM_001795 | CDH5 | Cadherin 5. type 2 (vascular endothelium) |
| NM_030582 | COL18A1 | Collagen. type XVIII. alpha 1 |
| NM_000091 | COL4A3 | Collagen. type IV. alpha 3 (Goodpasture antigen) |
| NM_001901 | CTGF | Connective tissue growth factor |
| NM_001511 | CXCL1 | Chemokine (C-X-C motif) ligand 1 (melanoma growth stimulating activity. alpha) |
| NM_001565 | CXCL10 | Chemokine (C-X-C motif) ligand 10 |
| NM_002994 | CXCL5 | Chemokine (C-X-C motif) ligand 5 |
| NM_002993 | CXCL6 | Chemokine (C-X-C motif) ligand 6 (granulocyte chemotactic protein 2) |
| NM_002416 | CXCL9 | Chemokine (C-X-C motif) ligand 9 |
| NM_001955 | EDN1 | Endothelin 1 |
| NM_182685 | EFNA1 | Ephrin-A1 |
| NM_004093 | EFNB2 | Ephrin-B2 |
| NM_001963 | EGF | Epidermal growth factor |
| NM_000118 | ENG | Endoglin |
| NM_004444 | EPHB4 | EPH receptor B4 |
| NM_004448 | ERBB2 | V-erb-b2 receptor tyrosine kinase 2 |
| NM_001993 | F3 | Coagulation factor III (thromboplastin. tissue factor) |
| NM_000800 | FGF1 | Fibroblast growth factor 1 (acidic) |
| NM_002006 | FGF2 | Fibroblast growth factor 2 (basic) |
| NM_000142 | FGFR3 | Fibroblast growth factor receptor 3 |
| NM_004469 | FIGF | C-fos induced growth factor (vascular endothelial growth factor D) |
| NM_002019 | FLT1 | Fms-related tyrosine kinase 1 (VEGFR1) |
| NM_002026 | FN1 | Fibronectin 1 |
| NM_000601 | HGF | Hepatocyte growth factor (hepapoietin A; scatter factor) |
| NM_001530 | HIF1A | Hypoxia inducible factor 1. alpha subunit (basic helix-loop-helix transcription factor) |
| NM_006665 | HPSE | Heparanase |
| NM_002165 | ID1 | Inhibitor of DNA binding 1. dominant negative helix-loop-helix protein |
| NM_024013 | IFNA1 | Interferon. alpha 1 |
| NM_000619 | IFNG | Interferon. gamma |
| NM_000618 | IGF1 | Insulin-like growth factor 1 (somatomedin C) |
| NM_000576 | IL1B | Interleukin 1. beta |
| NM_000600 | IL6 | Interleukin 6 (interferon. beta 2) |
| NM_000584 | IL8 | Interleukin 8 |
| NM_002210 | ITGAV | Integrin. alpha V (vitronectin receptor. alpha polypeptide. antigen CD51) |
| NM_000212 | ITGB3 | Integrin. beta 3 (platelet glycoprotein IIIa. antigen CD61) |
| NM_000214 | JAG1 | Jagged 1 |
| NM_002253 | KDR | Kinase insert domain receptor (a type III receptor tyrosine kinase) |
| NM_007015 | LECT1 | Leukocyte cell derived chemotaxin 1 |
| NM_000230 | LEP | Leptin |
| NM_002391 | MDK | Midkine (neurite growth-promoting factor 2) |
| NM_004995 | MMP14 | Matrix metallopeptidase 14 (membrane-inserted) |
| NM_004530 | MMP2 | Matrix metallopeptidase 2 (gelatinase A. 72kDa gelatinase. 72kDa type IV collagenase) |
| NM_004994 | MMP9 | Matrix metallopeptidase 9 (gelatinase B. 92kDa gelatinase. 92kDa type IV collagenase) |
| NM_000603 | NOS3 | Nitric oxide synthase 3 (endothelial cell) |
| NM_004557 | NOTCH4 | Notch 4 |
| NM_003873 | NRP1 | Neuropilin 1 |
| NM_003872 | NRP2 | Neuropilin 2 |
| NM_002607 | PDGFA | Platelet-derived growth factor alpha polypeptide |
| NM_000442 | PECAM1 | Platelet/endothelial cell adhesion molecule |
| NM_002619 | PF4 | Platelet factor 4 |
| NM_002632 | PGF | Placental growth factor |
| NM_002658 | PLAU | Plasminogen activator. urokinase |
| NM_000301 | PLG | Plasminogen |
| NM_021935 | PROK2 | Prokineticin 2 |
| NM_000962 | PTGS1 | Prostaglandin-endoperoxide synthase 1 (prostaglandin G/H synthase and cyclooxygenase) |
| NM_001400 | S1PR1 | Sphingosine-1-phosphate receptor 1 |
| NM_000602 | SERPINE1 | Serpin peptidase inhibitor. clade E (nexin. plasminogen activator inhibitor type 1). member 1 |
| NM_002615 | SERPINF1 | Serpin peptidase inhibitor. clade F (alpha-2 antiplasmin. pigment epithelium derived factor). member 1 |
| NM_021972 | SPHK1 | Sphingosine kinase 1 |
| NM_000459 | TEK | TEK tyrosine kinase. endothelial |
| NM_003236 | TGFA | Transforming growth factor. alpha |
| NM_000660 | TGFB1 | Transforming growth factor. beta 1 |
| NM_003238 | TGFB2 | Transforming growth factor. beta 2 |
| NM_004612 | TGFBR1 | Transforming growth factor. beta receptor 1 |
| NM_003246 | THBS1 | Thrombospondin 1 |
| NM_003247 | THBS2 | Thrombospondin 2 |
| NM_005424 | TIE1 | Tyrosine kinase with immunoglobulin-like and EGF-like domains 1 |
| NM_003254 | TIMP1 | TIMP metallopeptidase inhibitor 1 |
| NM_003255 | TIMP2 | TIMP metallopeptidase inhibitor 2 |
| NM_000362 | TIMP3 | TIMP metallopeptidase inhibitor 3 |
| NM_000594 | TNF | Tumor necrosis factor |
| NM_001953 | TYMP | Thymidine phosphorylase |
| NM_003376 | VEGFA | Vascular endothelial growth factor A |
| NM_003377 | VEGFB | Vascular endothelial growth factor B |
| NM_005429 | VEGFC | Vascular endothelial growth factor C |

* Human angiogenesis RT² Profiler™ PCR array (Catalog number PAHS-024Z, SABiosciences/Qiagen). **NCBI reference sequence database.
