# Supplementary material for: Functional intratumoral lymphatics in patient-derived xenograft models of squamous cell carcinoma of the uterine cervix: implications for lymph node metastasis
Source: Oncotarget. 2016 Jul 29;7(35):56986–97. doi: 10.18632/oncotarget.10931 (PMC5302967; doi:10.18632/oncotarget.10931)
Supplement: Supplementary file 3 [file oncotarget-07-56986-s003.docx]

**Supplementary Table S2.** Fold difference in gene expression between donor patient’s tumor (DPT) and patient-derived xenograft (PDX) model

| **Gene*** | **Expression ratio (DPT/PDX)** | | | |
| --- | --- | --- | --- | --- |
|  | **BK-12** | **ED-15** | **HL-16** | **LA-19** |
| AKT1 | 1.004 | 0.839 | 0.787 | 1.623 |
| ANG | 1.008 | 1.532 | 2.414 | 0.347 |
| ANGPT1 | 0.093 | 0.432 | 2.122 | 1.793 |
| ANGPT2 | 0.264 | 0.488 | 0.971 | 3.425 |
| ANGPTL4 | 4.160 | 1.649 | 4.942 | 3.458 |
| ANPEP | 2.672 | 0.575 | 2.477 | 2.350 |
| BAI1 | 0.885 | 1.047 | 0.947 | 2.140 |
| CCL11 | 1.886 | 1.696 | 2.949 | 6.661 |
| CCL2 | 0.964 | 3.137 | 0.793 | 2.731 |
| CDH5 | 0.822 | 0.896 | 2.652 | 1.736 |
| COL18A1 | 0.281 | 1.085 | 0.967 | 2.559 |
| COL4A3 | 0.214 | 1.106 | 1.695 | 5.740 |
| CTGF | 7.173 | 1.809 | 15.132 | 3.542 |
| **CXCL1** | **5.638** | **12.908** | **25.901** | **14.403** |
| CXCL10 | 0.122 | 3.020 | 0.917 | 0.053 |
| CXCL5 | 0.465 | 1.044 | 2.383 | 1.716 |
| CXCL6 | 1.047 | 1.696 | 5.207 | 6.661 |
| CXCL9 | 0.268 | 1.904 | 1.722 | 1.846 |
| EDN1 | 8.431 | 4.885 | 7.880 | 2.825 |
| EFNA1 | 9.696 | 1.434 | 10.156 | 1.176 |
| EFNB2 | 1.954 | 2.017 | 2.478 | 0.673 |
| EGF | 0.370 | 0.600 | 3.275 | 3.639 |
| ENG | 2.585 | 2.874 | 2.464 | 1.433 |
| EPHB4 | 0.585 | 0.669 | 0.897 | 1.743 |
| ERBB2 | 0.812 | 0.329 | 0.820 | 0.803 |
| F3 | 1.381 | 1.585 | 11.367 | 0.529 |
| FGF1 | 0.584 | 1.083 | 2.949 | 0.922 |
| FGF2 | 0.253 | 0.242 | 0.530 | 0.503 |
| FGFR3 | 1.749 | 0.937 | 1.500 | 2.706 |
| FIGF | 0.396 | 0.439 | 1.186 | 8.255 |
| FLT1 | 0.463 | 0.231 | 2.949 | 6.661 |
| FN1 | 0.424 | 1.942 | 1.459 | 0.344 |
| HGF | 1.698 | 1.696 | 2.949 | 6.661 |
| HIF1A | 1.504 | 1.018 | 0.822 | 0.802 |
| HPSE | 0.568 | 1.082 | 10.197 | 2.017 |
| ID1 | 1.121 | 1.658 | 1.705 | 1.724 |
| IFNA1 | 0.184 | 0.520 | 0.962 | 0.426 |
| IFNG | 1.698 | 1.696 | 2.949 | 6.661 |
| IGF1 | 0.351 | 0.554 | 0.805 | 2.201 |
| IL1B | 7.295 | 4.355 | 12.048 | 0.970 |
| IL6 | 15.589 | 8.570 | 5.965 | 3.245 |
| **IL8** | **19.693** | **85.782** | **133.515** | **10.981** |
| ITGAV | 1.022 | 0.956 | 1.754 | 1.210 |
| ITGB3 | 0.456 | 0.448 | 7.021 | 8.222 |
| JAG1 | 1.209 | 1.707 | 3.369 | 2.317 |
| KDR | 1.575 | 0.140 | 2.949 | 5.792 |
| **LECT1** | **6.153** | **5.363** | **10.396** | **6.661** |
| LEP | 0.255 | 0.358 | 1.861 | 2.141 |
| MDK | 0.241 | 0.486 | 0.778 | 0.315 |
| MMP14 | 0.520 | 0.513 | 0.920 | 2.819 |
| MMP2 | 0.229 | 1.393 | 13.173 | 3.427 |
| MMP9 | 0.260 | 1.546 | 4.106 | 0.152 |
| NOS3 | 0.233 | 0.098 | 0.283 | 1.653 |
| NOTCH4 | 1.504 | 4.580 | 3.923 | 1.683 |
| NRP1 | 0.413 | 0.751 | 0.529 | 0.653 |
| NRP2 | 0.631 | 1.195 | 5.393 | 2.739 |
| PDGFA | 1.369 | 2.126 | 1.298 | 2.357 |
| PECAM1 | 1.230 | 1.696 | 4.270 | 6.661 |
| PF4 | 1.651 | 1.696 | 10.677 | 5.591 |
| PGF | 2.741 | 0.965 | 1.689 | 2.406 |
| PLAU | 1.358 | 1.411 | 3.110 | 2.464 |
| PLG | 0.037 | 0.018 | 0.040 | 0.550 |
| PROK2 | 2.224 | 1.696 | 2.949 | 3.149 |
| PTGS1 | 0.390 | 0.512 | 1.292 | 0.374 |
| S1PR1 | 0.313 | 0.204 | 5.996 | 1.048 |
| SERPINE1 | 1.691 | 1.258 | 9.503 | 2.234 |
| SERPINF1 | 0.738 | 1.650 | 2.463 | 0.762 |
| SPHK1 | 0.658 | 1.055 | 4.372 | 2.622 |
| TEK (Tie-2) | 0.243 | 0.333 | 2.850 | 0.534 |
| TGFA | 1.048 | 0.471 | 2.521 | 2.515 |
| TGFB1 | 1.903 | 0.974 | 2.466 | 3.118 |
| TGFB2 | 0.820 | 1.774 | 1.491 | 0.681 |
| TGFBR1 | 0.736 | 0.791 | 1.035 | 1.141 |
| THBS1 | 1.015 | 1.173 | 1.928 | 1.170 |
| THBS2 | 0.373 | 0.159 | 2.084 | 1.381 |
| TIE1 | 1.507 | 1.696 | 2.949 | 4.644 |
| TIMP1 | 0.473 | 0.728 | 0.282 | 0.708 |
| TIMP2 | 0.292 | 0.460 | 1.254 | 1.072 |
| TIMP3 | 0.845 | 0.803 | 0.708 | 0.645 |
| **TNF** | **19.927** | **5.674** | **46.143** | **5.857** |
| TYMP | 0.526 | 0.548 | 0.416 | 0.929 |
| VEGFA | 4.030 | 1.254 | 3.138 | 1.702 |
| VEGFB | 0.993 | 0.978 | 1.136 | 0.656 |
| VEGFC | 0.397 | 0.396 | 10.346 | 0.792 |

* Six genes showed >2-fold higher expression in all DPTs than in the corresponding PDX model (marked red) and four of these genes showed >5-fold higher expression in all DPTs than in the corresponding PDX model (marked bold red).
