# Supplementary material for: Functional intratumoral lymphatics in patient-derived xenograft models of squamous cell carcinoma of the uterine cervix: implications for lymph node metastasis
Source: Oncotarget. 2016 Jul 29;7(35):56986–97. doi: 10.18632/oncotarget.10931 (PMC5302967; doi:10.18632/oncotarget.10931)
Supplement: Supplementary file 4 [file oncotarget-07-56986-s004.docx]

**Supplementary Table S3.** Fold difference in gene expression between highly metastatic (BK-12, LA-19) and poorly/non-metastatic (ED-15, HL-16) PDX models

| **Gene*** | **BK-12/ED-15** | **BK-12/HL-16** | **LA-19/ED-15** | **LA-19/HL-16** |
| --- | --- | --- | --- | --- |
| AKT1 | 1.842 | 1.804 | 1.205 | 1.181 |
| ANG | 3.270 | 8.786 | 1.436 | 3.857 |
| ANGPT1 | 1.873 | 8.420 | 0.382 | 1.718 |
| ANGPT2 | 1.157 | 0.554 | 0.718 | 0.344 |
| ANGPTL4 | 0.419 | 0.126 | 0.648 | 0.195 |
| ANPEP | 0.734 | 0.200 | 0.663 | 0.181 |
| BAI1 | 4.733 | 4.586 | 0.652 | 0.632 |
| CCL11 | 0.833 | 0.925 | 0.838 | 0.931 |
| CCL2 | 9.180 | 0.548 | 2.904 | 0.173 |
| CDH5 | 0.884 | 1.670 | 1.700 | 3.213 |
| COL18A1 | 1.738 | 22.205 | 0.293 | 3.739 |
| COL4A3 | 4.494 | 0.463 | 0.605 | 0.062 |
| CTGF | 1.339 | 11.082 | 8.194 | 67.837 |
| CXCL1 | 23.687 | 2.783 | 1.736 | 0.204 |
| **CXCL10** | **17351.225** | **109.889** | **1286.859** | **8.150** |
| CXCL5 | 2.551 | 3.335 | 2.004 | 2.619 |
| CXCL6 | 4.991 | 5.544 | 0.838 | 0.931 |
| CXCL9 | 13.669 | 8.582 | 3.024 | 1.899 |
| EDN1 | 1.429 | 7.125 | 0.744 | 3.710 |
| EFNA1 | 0.060 | 0.596 | 0.046 | 0.459 |
| EFNB2 | 1.370 | 25.373 | 0.872 | 16.143 |
| EGF | 1.978 | 0.595 | 0.318 | 0.096 |
| ENG | 1.116 | 0.425 | 107.972 | 41.082 |
| EPHB4 | 2.820 | 1.481 | 2.936 | 1.542 |
| ERBB2 | 0.444 | 0.530 | 0.174 | 0.207 |
| **F3** | **5.695** | **39.255** | **44.242** | **304.933** |
| FGF1 | 0.030 | 23.849 | 0.028 | 22.279 |
| FGF2 | 1.073 | 2.457 | 5.489 | 12.571 |
| FGFR3 | 1.098 | 0.750 | 0.206 | 0.141 |
| FIGF | 1.086 | 0.487 | 0.229 | 0.103 |
| FLT1 | 0.454 | 3.697 | 0.114 | 0.931 |
| **FN1** | **74.437** | **95.821** | **1034.506** | **1331.699** |
| HGF | 0.833 | 0.925 | 0.838 | 0.931 |
| HIF1A | 1.165 | 0.240 | 0.591 | 0.122 |
| HPSE | 1.943 | 84.047 | 2.080 | 89.977 |
| ID1 | 0.871 | 2.606 | 0.231 | 0.690 |
| IFNA1 | 2.208 | 2.092 | 2.598 | 2.461 |
| IFNG | 0.833 | 0.925 | 0.838 | 0.931 |
| IGF1 | 3.029 | 2.237 | 4.147 | 3.062 |
| IL1B | 15.361 | 4.437 | 46.353 | 13.390 |
| IL6 | 358.330 | 3.477 | 22.620 | 0.220 |
| IL8 | 29.206 | 15.539 | 1.935 | 1.029 |
| ITGAV | 0.707 | 0.781 | 1.044 | 1.153 |
| ITGB3 | 3.702 | 7.276 | 22.952 | 45.108 |
| JAG1 | 0.900 | 1.715 | 1.380 | 2.632 |
| KDR | 0.075 | 1.001 | 0.080 | 1.071 |
| LECT1 | 1.503 | 1.580 | 0.838 | 0.881 |
| LEP | 1.979 | 1.070 | 1.172 | 0.633 |
| MDK | 2.772 | 1.089 | 2.334 | 0.917 |
| MMP14 | 0.820 | 0.485 | 2.989 | 1.768 |
| MMP2 | 0.282 | 74.962 | 0.111 | 29.420 |
| MMP9 | 30.290 | 1592.244 | 4.450 | 233.937 |
| NOS3 | 2.569 | 1.734 | 1.605 | 1.084 |
| NOTCH4 | 1.347 | 1.665 | 0.924 | 1.143 |
| NRP1 | 0.664 | 2.512 | 0.121 | 0.459 |
| NRP2 | 1.952 | 95.126 | 1.105 | 53.813 |
| PDGFA | 0.298 | 0.737 | 0.677 | 1.672 |
| PECAM1 | 1.118 | 0.948 | 0.838 | 0.711 |
| PF4 | 0.833 | 0.318 | 1.989 | 0.759 |
| PGF | 0.571 | 0.182 | 1.338 | 0.427 |
| PLAU | 0.207 | 1.371 | 1.203 | 7.956 |
| PLG | 0.420 | 0.594 | 0.107 | 0.152 |
| PROK2 | 0.887 | 0.985 | 1.773 | 1.970 |
| PTGS1 | 0.361 | 0.169 | 0.106 | 0.049 |
| S1PR1 | 5.359 | 3.760 | 9.361 | 6.568 |
| SERPINE1 | 0.728 | 3.599 | 3.763 | 18.594 |
| SERPINF1 | 1.043 | 2.394 | 1.035 | 2.374 |
| SPHK1 | 1.007 | 0.293 | 2.414 | 0.702 |
| TEK (Tie-2) | 2.568 | 4.763 | 3.368 | 6.248 |
| TGFA | 1.001 | 2.813 | 3.044 | 8.552 |
| TGFB1 | 0.164 | 7.375 | 0.452 | 20.339 |
| TGFB2 | 4.046 | 24.902 | 0.244 | 1.504 |
| TGFBR1 | 0.557 | 1.053 | 0.817 | 1.545 |
| THBS1 | 1.244 | 0.281 | 6.137 | 1.386 |
| THBS2 | 4.012 | 6.135 | 27.607 | 42.209 |
| TIE1 | 0.938 | 1.042 | 6.078 | 6.751 |
| TIMP1 | 2.352 | 12.510 | 13.068 | 69.523 |
| TIMP2 | 1.436 | 51.920 | 37.709 | 1363.128 |
| TIMP3 | 0.249 | 40.733 | 2.301 | 376.510 |
| TNF | 19.000 | 4.675 | 36.551 | 8.993 |
| TYMP | 10.133 | 1.365 | 0.593 | 0.080 |
| VEGFA | 1.415 | 0.661 | 1.362 | 0.636 |
| VEGFB | 1.921 | 1.612 | 2.356 | 1.977 |
| **VEGFC** | **15.555** | **8.940** | **342.594** | **196.907** |

* Fifteen genes showed >2-fold higher expression in the BK-12 and LA-19 models than in the ED-15 and HL-16 models (marked red) and four of these genes showed >5-fold higher expression in the BK-12 and LA-19 models than in the ED-15 and HL-16 models (marked bold red). Only one gene showed >2-fold higher expression in the ED-15 and HL-16 models than in the BK-12 and LA-19 models (marked blue).
