# Supplementary material for: Functional intratumoral lymphatics in patient-derived xenograft models of squamous cell carcinoma of the uterine cervix: implications for lymph node metastasis
Source: Oncotarget. 2016 Jul 29;7(35):56986–97. doi: 10.18632/oncotarget.10931 (PMC5302967; doi:10.18632/oncotarget.10931)
Supplement: Supplementary file 5 [file oncotarget-07-56986-s005.docx]

**Supplementary Table S4.** Genes with expression levels that differed between BK-12-E and BK-12-L tumors by a factor of at least 2*

| **Gene**** | **Expression level** | | **BK-12-E/BK-12-L** | **Significance level** |
| --- | --- | --- | --- | --- |
|  | **BK-12-E** | **BK-12-L** |  |  |
| ANG | 7.91E-03 | 2.08E-02 | 0.380 | *P* < 0.005 |
| ANGPTL4 | 4.78E-03 | 1.02E-02 | 0.469 | *P* < 0.05 |
| CXCL1 | 1.69E-01 | 3.77E-02 | 4.472 | *P* < 0.005 |
| CXCL10 | 2.67E-01 | 5.72E-02 | 4.659 | *P* < 0.001 |
| CXCL6 | 7.67E-05 | 2.76E-05 | 2.774 | *P* < 0.05 |
| F3 | 1.34E-01 | 6.71E-02 | 2.002 | *P* < 0.05 |
| FGF1 | 3.30E-04 | 9.27E-04 | 0.356 | *P* < 0.005 |
| FGF2 | 3.59E-03 | 1.46E-03 | 2.455 | *P* < 0.005 |
| FN1 | 2.68E-03 | 1.03E-03 | 2.610 | *P* < 0.05 |
| ID1 | 2.04E-01 | 4.41E-01 | 0.463 | *P* < 0.005 |
| IGF1 | 1.42E-02 | 6.39E-03 | 2.222 | *P* < 0.05 |
| IL1B | 9.76E-03 | 4.48E-03 | 2.179 | *P* < 0.05 |
| IL6 | 5.50E-03 | 1.75E-03 | 3.146 | *P* < 0.005 |
| NOS3 | 1.68E-03 | 8.35E-04 | 2.012 | *P* < 0.05 |
| PGF | 9.78E-03 | 2.35E-02 | 0.416 | *P* < 0.01 |
| PLG | 6.10E-04 | 2.76E-05 | 22.051 | *P* < 0.001 |
| S1PR1 | 1.74E-03 | 6.99E-04 | 2.488 | *P* < 0.05 |
| TEK (Tie-2) | 8.12E-04 | 4.01E-04 | 2.025 | *P* < 0.05 |
| TGFB1 | 1.13E-02 | 3.30E-02 | 0.342 | *P* < 0.05 |
| THBS1 | 4.59E-02 | 1.32E-02 | 3.477 | *P* < 0.001 |
| TIMP1 | 5.36E-03 | 1.98E-03 | 2.709 | *P* < 0.001 |
| TIMP3 | 1.87E-02 | 7.53E-02 | 0.248 | *P* < 0.05 |
| TYMP | 1.11E-01 | 5.41E-02 | 2.052 | *P* < 0.01 |
| VEGFC | 6.34E-03 | 2.72E-03 | 2.334 | *P* < 0.05 |

* BK-12-E, BK-12 tumors in passages 4-5. BK-12-L, BK-12 tumors in passages 15-20. ** Seventeen genes showed significantly higher expression in BK-12-E tumors than in BK-12-L tumors (marked red), and seven genes showed significantly higher expression in BK-12-L tumors than in BK-12-E tumors (marked blue).
